# Adverse events during rotary-wing transport of mechanically ventilated patients: a retrospective cohort study

**DOI:** 10.1186/cc6909

**Published:** 2008-05-22

**Authors:** Christopher W Seymour, Jeremy M Kahn, C William Schwab, Barry D Fuchs

**Affiliations:** 1 Division of Pulmonary and Critical Care, University of Washington School of Medicine, Campus Box 356522, Seattle, WA 98195-6522, USA; 2 Division of Pulmonary, Allergy and Critical Care, Leonard Davis Institute for Health Economics and the Center for Clinical Epidemiology and Biostatistics, University of Pennsylvania School of Medicine, University of Pennsylvania Medical Center, Blockley Hall, Room 723, 423 Guardian Drive, Philadelphia, PA 19104-6160, USA; 3 Division of Traumatology and Surgical Critical Care, PENNstar Flight, Hospital of the University of Pennsylvania, 3440 Market Street, First Floor, Philadelphia, PA 19104, USA; 4 Medical Intensive Care Unit and Respiratory Care Services, Division of Pulmonary, Allergy, and Critical Care Medicine, Hospital of the University of Pennsylvania, 3400 Spruce Street, Philadelphia, PA 19104, USA

## Abstract

**Introduction:**

Patients triaged to tertiary care centers frequently undergo rotary-wing transport and may be exposed to additional risk for adverse events. The incidence of physiologic adverse events and their predisposing factors in mechanically ventilated patients undergoing aeromedical transport are unknown.

**Methods:**

We performed a retrospective review of flight records of all interfacility, rotary-wing transports to a tertiary care, university hospital during 2001 to 2003. All patients receiving mechanical ventilation via endotracheal tube or tracheostomy were included; trauma, scene flights, and fixed transports were excluded. Data were abstracted from patient flight and hospital records. Adverse events were classified as either major (death, arrest, pneumothorax, or seizure) or minor (physiologic decompensation, new arrhythmia, or requirement for new sedation/paralysis). Bivariate associations between hospital and flight characteristics and the presence of adverse events were examined.

**Results:**

Six hundred eighty-two interfacility flights occurred during the period of review, with 191 patients receiving mechanical ventilation. Fifty-eight different hospitals transferred patients, with diagnoses that were primarily cardiopulmonary (45%) and neurologic (37%). Median flight distance and time were 42 (31 to 83) km and 13 (8 to 22) minutes, respectively. No major adverse events occurred during flight. Forty patients (22%) experienced a minor physiologic adverse event. Vasopressor requirement prior to flight and flight distance were associated with the presence of adverse events in-flight (*P *< 0.05). Patient demographics, time of day, season, transferring hospital characteristics, and ventilator settings before and during flight were not associated with adverse events.

**Conclusion:**

Major adverse events are rare during interfacility, rotary-wing transfer of critically ill, mechanically ventilated patients. Patients transferred over a longer distance or transferred on vasopressors may be at greater risk for minor adverse events during flight.

## Introduction

Aeromedical transport is widely used to transport critically ill patients between facilities. Patients may be transferred to receive a higher intensity of care, for specific procedures, or to maintain continuity between patients and clinicians familiar with their care [1]. Transferred patients may represent up to 20% of the admissions in tertiary care intensive care units (ICUs), are known to have a higher severity of illness compared with other ICU patients, and have worse-than-expected ICU outcomes [2-4].

Interhospital transport of severely ill patients is not without risk [5,6]. In prior heterogeneous cohorts of patients undergoing interfacility transfer, adverse events have occurred in up to 34% of patients [7]. Air transport may introduce even more risk, due to patient anxiety [8], movement of patients in smaller confines, or difficulty in performing advanced life support tasks by providers [9-11]. Though best described during intrahospital transport of critically ill patients, non-physiologic, equipment-related incidents may also pose a risk during flight [12]. The majority of data on rotary-wing transportation and adverse events are derived from short-distance transfer of cardiac patients, triaged during the early course of acute myocardial infarction [13,14], as well as trauma, burn, and pediatric cohorts [15-17]. However, the overall incidence of adverse events during interfacility, aeromedical transport of mechanically ventilated patients remains unknown [6]. Additionally, some specialty organizations have called for the development of a tiered, regionalized system of critical care, which potentially could increase the need for interhospital transport [18]. If a regionalized system of critical care is to be considered, it is imperative to establish the safety of routine interfacility, aeromedical transport. The purpose of this study was to determine the frequency of and factors associated with adverse physiologic events during aeromedical transport of mechanically ventilated patients during interfacility transfers to a tertiary care, university hospital.

## Materials and methods

### Study design and patients

We conducted a retrospective cohort study of patients transferred to the Hospital of the University of Pennsylvania (HUP) by rotary-wing transport from June 2001 to June 2003. The HUP is a 685-bed, university-affiliated, tertiary care hospital with 92 ICU beds serving the greater Philadelphia area, including southeastern Pennsylvania and parts of Delaware, New York, Maryland, and New Jersey. PENNstar Flight provides all the aeromedical transport for critically ill patients transferred from referring hospitals to the HUP. Flight crews consist of a paramedic, nurse, pilot, and additional crew if space allows (physician, nurse, or paramedic). No physicians were present on flights during the study period; however, a medical command physician was available to provide consultations and treatment guidance to referring institutions. All patients transported via PENNstar Flight from a referring hospital and admitted to an HUP ICU were eligible for the study. We excluded transports from trauma scenes, patients transferred from the HUP to other facilities, patients referred for transfer but not flown, and fixed-wing flights. The Investigational Review Board of the HUP approved this research protocol with a waiver of written informed consent.

### Variables

We obtained computerized flight records from the central PENNstar database. Discharge records from the HUP were linked with the PENNstar dataset to determine patient outcomes. Flight records were reviewed to abstract demographic data, reason for transfer, initial vital signs, and ventilator settings encountered by the PENNstar crew at the time of arrival at the transferring facility. In-flight data included flight personnel, vital signs (documented every 5 minutes by the flight crew), and all ventilator or airway manipulations, type and presence of intravenous access, and use of medications such as vasopressors, sedatives, and neuromuscular blockade.

The primary outcome variable was in-flight adverse events, categorized as either major or minor. Based upon on our review of the literature, there is no consensus or consistency in the definitions used to describe major and minor adverse events [7,19-21]. Thus, similar to other reports, we defined major adverse events during interhospital transport as death, cardiac/respiratory arrest, pneumothorax, and seizure. Minor adverse events were defined as (a) respiratory: new arterial oxygen saturation (SaO_2_) of less than 85%, decrease in SaO_2 _by 10%, or ventilator change during flight (including transition to manual ventilation); (b) cardiovascular: new mean arterial pressure of less than 60 mm Hg, new heart rate of less than 60 beats per minute, change in heart rate or mean arterial pressure requiring medication administration, or new arrhythmia; or (c) administration of sedative or neuromuscular blockade for a change in vital signs or ventilator dysynchrony.

Transferring hospital characteristics were obtained from the 2003 American Medical Association annual survey. Data abstracted included hospital ownership (profit or non-profit), community size (large urban, small urban, or rural), number of hospital and ICU beds, and landing zone location. The time of flight, date, duration (in minutes), and distance (in kilometers) were also determined for each patient. Arc distances between hospitals were calculated using the exact latitude and longitude of each facility [22]. Patient outcomes, including ICU and hospital lengths of stay, ventilator days, and discharge status, were determined from the hospitals' administrative database in evaluable patients.

### Statistical analysis

Values are presented as mean ± standard deviation, median (interquartile range), or frequency (percentage). Bivariate associations between hospital and flight characteristics and the presence of adverse events were evaluated using an unpaired *t *test, the Wilcoxon rank sum test, or a chi-square test, as appropriate. Because the small number of adverse events limited model size and because we were not interested in the effect of exposures independent of other variables, a multivariate analysis was not performed. Statistical analyses were performed using Stata 9.0 (StataCorp LP, College Station, TX, USA). All tests were two-tailed, and a *P *value of 0.05 was considered significant.

## Results

**Figure 1:**
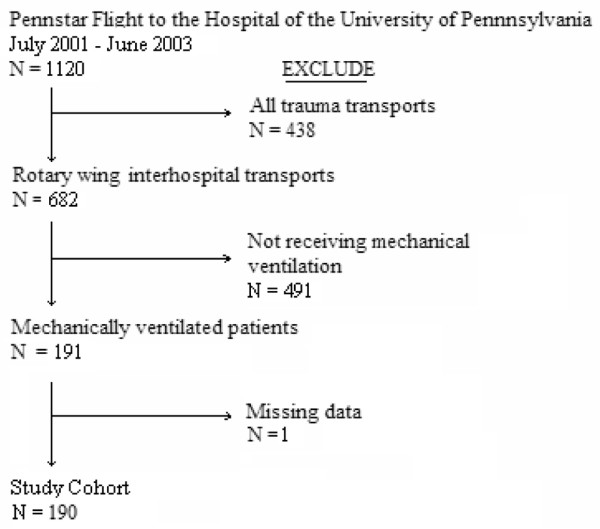
Diagram of patient accrual.

During the 36-month period of review, 1,120 patients were transferred by PENNstar Flight to the HUP. One hundred ninety-one were rotary-wing, interfacility, aeromedical transports of patients who were receiving mechanical ventilation at the time of flight (one patient had no data available for review) (Figure 1). Patient characteristics are shown in Table 1 and diagnoses are presented in Table 2. Median flight distance was 48 (31 to 83) km, with the majority of flights during daylight hours (7 a.m. to 7 p.m.). Mode of ventilation was primarily volume-cycled via endotracheal tube, with a large proportion of patients receiving vasopressors (29%) and neuromuscular blockade from the transferring hospital or flight staff prior to flight (54%). Sixteen percent of patients had the ventilator mode changed by flight staff prior to flight, and 17% had an increase in fraction of inspired oxygen (median increase = 0.5 [0.4 to 0.6]). Fifty-eight hospitals transferred patients during the period of review (Table 3). Transferring hospitals were located primarily in large urban areas. Many hospitals did not have a landing zone located at the hospital (51%).

**Table 1:** Patient characteristics (n = 190)

Demographics	
Age, years	55 ± 16
Female gender, number (percentage)	85 (45)
Transferring hospital patient data	
Glasgow Coma Scale score	5 (3–9)
Length of stay, days	1 (1–3)
Location	
Intensive care unit	113 (60)
Emergency room	76 (40)
Other	1 (<1)
Vital signs	
Heart rate, beats per minute	98 ± 24
Respiratory rate, breaths per minute	17 ± 5
Mean arterial pressure, mm Hg	90 ± 22
SaO_2 _< 90%	11 (6)
Use of neuromuscular blockers prior to flight	101 (54)
Use of sedation prior to flight^a^	150 (84)
Use of vasopressors prior to flight	55 (29)
Transferring hospital ventilator data	
Mode	
Assist control	152 (80)
Intermittent mandatory ventilation	19 (10)
Pressure support ventilation	2 (1)
Pressure control ventilation	3 (2)
Other^b^	14 (7)
Settings	
Tidal volume, mL	654 ± 105
Positive end-expiratory pressure, cm H_2_O	5.4 ± 4.7
FiO_2_, percentage	0.73 ± 0.27
Tracheostomy	7 (4)
Flight data	
Nighttime transfer	76 (40)
Winter transfer	40 (21)
Distance, km	48 (31–83)
Flight time, minutes	13 (8–22)
In-flight ventilator data	
Initial mode	
Assist control	147 (77)
Intermittent mandatory ventilation	14 (7)
Manual ventilation^c^	25 (13)
T-piece	2 (1)
Not recorded	2 (1)
FiO_2_, percentage	0.84 ± 0.25
Tidal volume, mL	662 ± 124
Receiving hospital outcomes^d^	
Duration of mechanical ventilation, days	6 (3–18)
Intensive care unit length of stay, days	11 (3–21)
Hospital length of stay, days	15 (6–31)
Hospital mortality, percentage	42 (31)

Values are presented as number (percentage), mean ± standard deviation, or median (interquartile range), as appropriate. ^a^Pre-flight sedation includes benzodiazepines, propofol, and opiates; data are missing for 9 patients. ^b^Includes airway pressure release, pressure-regulated volume controlled, and manual ventilation. ^c^Includes patients transitioned to manual ventilation during flight. ^d^Outcome data are not available from the receiving hospital in 53 of 190 patients (28%). FiO_2_, fraction of inspired oxygen; SaO_2_, oxygen saturation.

**Table 2:** Patient diagnoses at the time of transfer

Cardiology and cardiac surgery		45 (24)
Acute coronary syndrome without shock	9 (20)	
Cardiogenic shock	20 (45)	
Thoracic/abdominal aortic aneurysm	4 (9)	
Post-cardiac arrest	8 (18)	
Other	4 (9)	
Neurological		70 (37)
Cerebral vascular accident	15 (21)	
Intracerebral hemorrhage	54 (77)	
Meningitis	1 (1)	
Pulmonary		40 (21)
Asthma/Chronic obstructive pulmonary disease	4 (10)	
Hemopytsis	3 (8)	
Respiratory failure	29 (72)	
Other	4 (10)	
Surgical		10 (5)
Traumatic injury	8 (80)	
Necrotizing fasciitis	2 (20)	
Drug overdose/Poisoning		9 (5)
Gastrointestinal		9 (5)
Bleeding	4 (44)	
Liver failure	5 (55)	
Oncology		6 (3)

Values are presented as number (percentage)

**Table 3:** Transferring hospital characteristics

Total referring hospitals, number	58
Ownership	
Non-profit	54 (93)
For-profit	4 (7)
Government	0 (0)
Total beds, number	300 ± 177
Intensive care unit beds, number	25 ± 14
Trauma center	14 (24)
Off-site landing zone for rotary-wing pick-up^a^	29 (51)
Community size	
Rural	2 (4)
Small urban	21 (36)
Large urban	35 (60)
Affiliated with a medical school	27 (47)

Values are presented as number (percentage) or mean ± standard deviation. ^a^Data are missing for one hospital.

**Figure 2:**
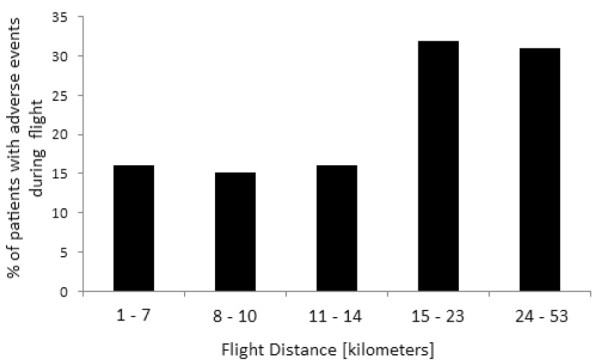
Presence of minor physiologic events during flight, stratified according to quintile of flight distance (kilometers).

Adverse events were uncommon during flight (Table 4). No major adverse events, including death, cardiac arrest, or pneumothorax, occurred during transport. Minor events were more frequent (22% of patients), and the administration of neuromuscular blockade/sedation or ventilator change for an alteration in vital signs was the most common. Administration of beta blockers, adjustment of vasopressors, and fluid boluses were the most common medicines administered during flight. Table 5 shows patient and flight characteristics categorized by the presence (n = 40) or absence (n = 140) of adverse events during flight. Only the presence of vasopressors and flight distance were associated with adverse events (*P *< 0.05). Vasopressor use was more common in patients transported from transferring hospital ICUs compared with emergency rooms (35% versus 18%; *P *< 0.01). Patients' demographics, level of ventilator support, use of manual ventilation, presenting vital signs, transferring hospital characteristics, and season were not significantly associated with adverse events. Quintiles of flight distance are shown in Figure 2; the incidence of adverse events is stable in the first three quintiles and increases in the highest two quintiles.

**Table 4:** Physiologic adverse events during interfacility transport of 190 patients

Major adverse events	
Death	0 (0)
Cardiovascular collapse requiring advanced cardiac life support	0 (0)
Seizure	0 (0)
Pneumothorax	0 (0)
Minor adverse events^a^	
Respiratory compromise	
New oxygen saturation <85%	7 (4)
Oxygen saturation decrease by >10%^b^	8 (4)
In-flight change in ventilator settings^c^	4 (2)
Cardiovascular compromise	
New mean arterial pressure <60 mm Hg	5 (3)
New heart rate <60 beats per minute	4 (2)
New arrhythmia	3 (2)
New administration of medicine for compromise of vital signs^d^	17 (9)
Administration of sedation/paralysis for vital sign change or ventilator dysynchrony	15 (8)

Values are presented as number (percentage). ^a^For 180 patients with complete physiologic data during flight. ^b^If normoxemic, oxygen saturation of greater than 90% before flight. ^c^Ventilator interventions included switching to manual ventilation or beginning inhaled prostacyclin. ^d^Includes new administration of fluid bolus, beta blockers, or titration of vasopressors.

**Table 5:** Bivariate analysis of transport variables and presence of adverse events^a^

Variable	Adverse event (n = 40)	No adverse event (n = 140)	*P *value
Patient characteristics			
Age, years	57 ± 16	55 ± 16	0.44
Female gender	43	43	0.97
Glasgow Coma Scale score	5 (3–8)	5 (3–9)	0.57
Outside hospital length of stay, days	1 (1–2)	1 (1–3)	0.52
Pre-flight presence of vasopressors, number (percentage)	18 (45)	30 (21)	<0.01
Pre-flight fraction of inspired oxygen, percentage	0.77 ± 27	0.72 ± 30	0.39
PEEP prior to flight	5 ± 5	6 ± 4	0.25
Patient in emergency room when transferred	39	37	0.84
Use of manual ventilation during flight, number (percentage)	5 (12.5)	17 (12)	1.0
Hospital characteristics			
Bed size			
<200	9 (26%)	38 (30%)	0.6
200–400	13 (37%)	53 (42%)	
>400	13 (37%)	36 (28%)	
Academic institution	15 (43%)	58 (46%)	0.77
Transfer characteristics			
Landing zone on site at transferring hospital	19 (49)	61 (46)	0.75
Nighttime transfer	15 (38%)	58 (41%)	0.66
Winter transfer	6 (15%)	33 (24%)	0.25
Distance, km	57 (35–95)	47 (31–82)	0.02
Flight time, minutes	15 (10–24)	12 (8–20)	0.32

Values are presented as number (percentage), mean ± standard deviation, or median (interquartile range), as appropriate. ^a^For 180 patients with complete physiologic data during flight. PEEP, positive end-expiratory pressure.

## Discussion

In this retrospective cohort study of mechanically ventilated patients transferred to a tertiary care medical center, rotary-wing aeromedical transport was safe, with a notable absence of major adverse events during flight. The presence of vasopressor use and longer flight distances were associated with an increased incidence of minor physiologic adverse events. These data are similar to findings of safety during ground transport of severely ill patients with respiratory failure [23] and should facilitate efforts to study systems that rely on interfacility transfer for regionalization of critical care [18].

A recent review of the literature emphasized the paucity of safety data available in patients during aeromedical transport [6]. The present cohort of intubated patients (n = 191) exceeds the total number of previously reported cases of ventilated patients undergoing interfacility, rotary-wing transfer [6,7,17,24]. Based upon prior reports of the safety of ground and fixed-wing transport, we hypothesized that major adverse events would be rare. We found that minor physiologic events occurred at a rate similar to those previously reported [7] and that major adverse events were absent. This mirrors the reports of others that death and cardiac arrest are uniformly uncommon (<2%) [7,8]. Vasopressor use was found to be associated with minor physiologic derangements, as well as a higher level of care at the time of transfer, likely reflecting an increased severity of illness and hemodynamic instability. Longer flight distance was also associated with minor physiologic adverse events, and surprisingly, this was not present for flight time. Both flight times and distance may increase patient risk simply due to longer exposure to inherent flight risks or may also be a marker of disease severity. In the absence of a uniform measurement of severity of illness from the outside hospitals, we were unable to assess whether patients transferred from distant hospitals were more ill. Unlike distance, which is a fixed variable, flight time may be associated with other unmeasured confounders such as weather and air speed [25].

This work may have implications for future studies of regionalization of care for critically ill patients. Recently, a task force comprised of the major critical care societies emphasized regionalization as an important strategy to increase patient access to tertiary care institutions with multidisciplinary delivery of critical care [26]. Similarly, the Prioritizing the Organization and Management of Intensive Care Services (PrOMIS) committee directly called for a tiered, regionalized system of adult critical care [18]. A large component of regionalization will be the routine transfer of critically ill, mechanically ventilated patients to tertiary care centers. If we are to duplicate the successful results of the regionalized trauma system [27], critical care regionalization will require accurate knowledge of the additive risk imposed during interfacility transfer and ways to predict patients at higher risk for transfer-related complications.

Our work has several limitations. Although 58 hospitals transferred patients, the cohort is limited to a single referral center, where all patients were transported by the PENNstar flight crew. Because not all technical complications (tubing, monitors, oxygen supply) were recorded, our analysis is limited to clinical events. These events were assessed in a retrospective fashion and were limited by the accuracy of data recorded by flight staff. As vital signs were documented in the record every 5 minutes, transient events may not have been captured. However, it is unlikely that any clinically significant event would not be noted by the flight crew, and free text recorded by flight staff in the flight record was also reviewed by study staff to search for these events which may have not been obtained by the vital sign record. Variability in the completeness of documentation of adverse events may have occurred due to differences in the flight crews; however, flight staff was composed of similarly trained individuals on all PENNstar flights. Also, the definitions employed for major and minor adverse events represent a consensus among investigators and recent literature, as no professional society has established uniform guidelines [7,19-21]. Discordant interpretation of various in-flight events as 'usual care' may further reduce the incidence of adverse events reported herein. The present cohort consists of short-distance, aeromedical transfer of patients and may not be generalizable to fixed-wing or longer distance rotary-wing flights (>100 km). Importantly, this cohort does not describe all patients undergoing interfacility transfer to tertiary care centers, as a certain proportion travel via ground ambulance [7,28]. The goal of this study was to evaluate the incidence of adverse events in aeromedical transport patients only as they are likely to be at the highest risk for complications. However, these patients were stabilized prior to interhospital transport at transferring facilities, perhaps reducing the incidence of major adverse events. Other data not evaluated were the duration of time during secondary ground transport in those patients delivered to a landing zone off-site from the transferring hospital (51%). This time may be a surrogate for severity of illness but may also represent an opportunity for equipment-related, non-physiologic adverse events [29]. Finally, the impact these in-flight adverse events may have on outcomes of subsequent care at tertiary care centers is not known, and the present work is notable for incomplete receiving hospital data. Multiple steps in patient care occur following rotary-wing transport and may include secondary transport to the receiving facility, re-triage in receiving hospital emergency rooms, definitive surgical interventions, new medical complications, and/or stabilization in destination ICUs [30]. It is established that transferred patients have worse ICU outcomes at receiving hospitals [2,30], and the present study was not designed to determine whether transfer events contribute to this finding.

## Conclusion

This cohort of mechanically ventilated patients undergoing interfacility, aeromedical transport confirms prior work in critically ill patients transferred via ground ambulance that major adverse events, including death, are rare. Minor physiologic events occur at a rate similar to those undergoing flight while not intubated and may be associated with concurrent use of vasopressors and longer flight distance. Prior to adoption of regionalized care systems, further multicenter, prospective studies are needed with consensus definitions of adverse events during flight and multivariate analysis of pre-flight risk factors.

## Key messages

• Transport of mechanically ventilated patients by rotary-wing transport is safe.

• Use of vasopressors and longer transport distance may be associated with minor adverse physiologic events during flight.

## Abbreviations

HUP = Hospital of the University of Pennsylvania; ICU = intensive care unit; SaO_2 _= oxygen saturation.

## Competing interests

The authors declare that they have no competing interests.

## Authors' contributions

CW Seymour assisted in study design, performed data acquisition, and drafted the manuscript. CW Schwab participated in study design and conceptualization and facilitated data acquisition. JK and BF helped to conceive the study, performed statistical analysis, and helped to draft the manuscript. All authors read and approved the final manuscript.
